# Harnessing community expertise in understanding food insecurity to inform responses in a local government area in Victoria, Australia: a mixed methods photovoice study

**DOI:** 10.1186/s12889-023-16796-0

**Published:** 2023-10-13

**Authors:** Annika Molenaar, Pieta Bucello, Sue Kleve

**Affiliations:** 1 Department of Nutrition, Dietetics and Food, School of Clinical Sciences, Monash University, Melbourne, VIC Australia; 2 Cardinia Shire Council, Melbourne, VIC Australia

**Keywords:** Food security, Food access, Photovoice, Local government, Community

## Abstract

**Background:**

Local food systems which support affordable, equitable, accessible, and sustainable food are important to enhance food access and reduce food insecurity. Cardinia Shire Council, a local government in Victoria, Australia has developed and endorsed a ‘Community Food Strategy’ to support their local food system and food security. This study aimed to explore local government community member perspectives regarding food access within their local food environment, and suggested areas to be addressed to better support access.

**Methods:**

A sequential mixed methods photovoice methodology was implemented. Participants aged over 18 years, who resided in Cardinia Shire, completed an online quantitative survey to explore demographics, food access and food security status and severity (18-item USDA Household Food Security Survey Module). The qualitative photovoice method was used, whereby participants were asked to take photographs that represent their experiences of food access. These photographs were used as prompts in a semi-structured interview Interview data were thematically analysed.

**Results:**

Seventeen participants completed the study, of which five participants experienced varied severity levels of food insecurity. From the photovoice interviews four themes were developed: 1) Food: a connector to self, people and place, 2) Influencers of food access and triggers for food insecurity, 3) Savvy food literacy skills to support access to food, 4) Consequences of and struggles with food insecurity. Participants suggested recommendations for action to support future food access in their community.

**Conclusions:**

While food choice is influenced by a range of determinants, the local food environment greatly impacts both food access and food choice. A supportive local food system which promotes inclusion of a community voice, community connectedness, food literacy and physical and economic access to local produce is crucial to support food security.

## Background

Access to food and food security are fundamental human rights. For food security to be achieved people will at all times, have physical, social and economic access to sufficient, safe and nutritious food to meet their food preferences, dietary and cultural needs for an active and healthy life [1]. Food security is underpinned by the six dimensions of: food availability, physical and economic access to food, utilisation of food including nutrient intake, agency, social and ecological sustainability [2] and the stability of all of these dimensions [3]. Disruptions in one or more of these dimensions negatively impacts food security status. At the household level the primary determinant of food insecurity is an inadequate and unstable income and financial resources. Additional factors also impact food security status such as affordable housing, household composition specifically sole-parent households, social isolation, education, and a sustainable local food system [4–10]. Food insecurity, is therefore not only due to a scarcity of food but is determined by a complex interaction of socio-environmental factors impacting food access at both a population, community, and household level.

Worldwide the prevalence of food insecurity is approximately 750 million people or nearly 10% of the global population [11]. Dependant on the measurement tool, estimates of the prevalence of household food insecurity in Australia are between 4 to 13% of the general population [12]. During the COVID-19 pandemic food insecurity increased, with estimates of up to 28% of Australian households experienced food insecurity, with many for the first time or of greater severity [13, 14]. Food insecurity has been linked to adverse physical and mental health and social outcomes. The impacts of social isolation and stress in those experiencing food insecurity have been associated with increased risk of mental illness [15]. Adults experiencing food insecurity have been found to consume less fruit, vegetables and dairy and have lower macro- and micronutrient intakes than adults who are food secure [16, 17]. Additionally, those experiencing food insecurity have been found to report poorer health with higher rates of chronic disease such as diabetes and hypertension [18, 19].

Food systems which support affordable, accessible, and sustainable food for all are important to support food security in Australia and worldwide. Australia’s food system is governed at multiple government levels for example national, state and local. The ability for local governments to act on food systems is constrained by both state and national political, legislative and financial factors [20]. The role of local governments include community planning and development, governance and regulation [21, 22], yet some local governments do not perceive food within their scope of responsibility, or lack the finances and resources to support food system policy and programs [20, 23]. Community perceptions of issues and their priority in turn influences local government’s political will to prioritise areas of work across these issues. If supported by state government policy and frameworks that promote public health, local governments can affect legislation and regulation to support public health; through measures such as urban planning and restricted development of fast food outlets [24]. However, current Australian state government planning legislation often does not consider public health, and therefore can hinder the power for local governments to make change [24]. Despite the absence of a national coordinated strategy amongst Australian governments to address food security, local governments are increasingly implementing strategies to address this issue. At the local government area (LGA), which encompass multiple geographically defined suburbs or localities administered by a local council organisation, strategies to address food security vary with mixed success and outcomes. Recent research in the Australian states of New South Wales and Victoria identified that the dominant response to food insecurity by 72% of local governments continues to be the provision of food relief or meals to vulnerable communities [25].

The LGA of Cardinia Shire is situated in the south-east fringe, 55 km from metropolitan Melbourne, Victoria, and includes more than 30 suburbs and townships with a forecasted population of 129,896 people as of 2023 [26–28]. Cardinia Shire has significant rural and horticultural land with agriculture, meat and food manufacturing being amongst the main industries [26]. It is classified as a growth LGA with a higher proportion of young families than the Melbourne average [27]. Additionally, culturally and linguistically diverse communities constitute a small proportion of the population [27]. It is ranked 40 out of 79 LGAs in Victoria for mortality rate and similar life expectancy to other LGAs in the surrounding area [29]. There is however, an increased lifetime risk from factors such as smoking, alcohol consumption and unhealthy eating amongst Cardinia Shire’s population and the rate of overweight and obesity (53.6%) is significantly higher than the Victorian average [29]. There is a lower proportion of high income earners that reside in Cardinia Shire than the Victorian average, and while the cost of housing is lower, there are more people experiencing mortgage and rental stress [29, 30]. The rates of car ownership are higher than the state average with a greater reliance on cars as the main form of transportation, often with a high commute time [29, 31]. The average cost of purchasing healthy food is higher than Victorian average and is approximately a third of the income for households relying on welfare payments, with food insecurity rates being estimated at 4% to 18% [29]. In 2021, using the validated United States Department of Agriculture Household Food Security Survey Module -18 item (USDA-HFSSM) [32] amongst a representative sample of households in Cardinia Shire, it was estimated that 21% of households are experiencing some form of food insecurity [33]. As part of the Victorian State Government legislative requirement of the Public Health and Wellbeing Act 2008, Cardinia Shire Council developed a Liveability Plan 2017–2029 as its Municipal Health and Wellbeing Plan.

This Liveability Plan 2017 − 2029 is a planning framework to ensure “Cardinia Shire is a liveable, resilient community where the environment flourishes and residents are healthy, included and connected” [29]. Food is recognised as a fundamental domain and as such Cardinia Shire implemented a ‘Community Food Strategy’ (2018—2026), which aims to promote a healthy, delicious, sustainable and fair food system across the Shire [34]. This strategy prioritises: affordable nutritious food, enhancing the local food system through utilising and protecting agricultural land, supporting food growers, increasing access to locally grown food, reducing and diverting food waste and enhancing food knowledge and skills in the community setting [34]. The strategy was developed using a community participatory policy making approach utilising ‘Kitchen Table’ conversations to explore challenges and solutions for the local food system through the lens of Cardinia Shire community members [35]. To gather additional perspectives, other community engagement activities were used such as digital forums and a campaign to promote the discussion of food systems within homes [35]. Findings from the community members as well as stakeholder workshops were used to shape the Cardinia Community Food Strategy, which is implemented across the LGA [35].

Since 2018, Cardinia Shire Council has partnered with Monash University in additional intelligence gathering research to further explore the prevalence and severity of food security, factors that impact on food security including food affordability and availability and perspectives of organisational stakeholders providing services to people experiencing food insecurity. Food security is complex, multidimensional and involves interactions between many socio-ecological and structural factors [36]. Access to food may be highly variable across localised areas and therefore it is important to consider the local food ecosystem and its potential effects on individuals within a community. To understand this, it is important to explore community lived experiences of the localised food system, factors that impact them and the implications for food access. This research aimed to understand Cardinia Shire community members' perspectives and experiences of accessing food within their local food environment and to identify areas to support access to nutritious food.

## Methods

### Study design

This study was grounded in pragmatism, where the researchers were interested in understanding participants perspectives and experiences of food access. A two-stage sequential mixed methods design, involving a quantitative online survey (part 1) and qualitative photovoice interviews (part 2) was employed. As the research aimed at understanding perspectives of the lived experiences accessing food by community members of Cardinia Shire, the focus of the study design was on the qualitative phase. To provide background understanding of participants prior to the interview, the quantitative online survey was used to gather demographic characteristics, determine food security status and factors related to food access. The results then informed prompts to guide the part 2 qualitative photovoice interviews.

Ethics was approved by the Monash University Human Research Ethics Committee (Approval number: 27023). Informed written consent was gathered for both parts of the study. Approval was received from participants to use photographs without distinguishing features, such as faces, in publications. Participant anonymity was maintained by assigning participant ID codes to survey responses and interview transcripts and use of pseudonyms in research outputs.

### Participants and setting

A cross-sectional convenience sample of participants were recruited from Cardinia Shire, Victoria, Australia. Participants were eligible if they currently lived in Cardinia Shire and were 18 years or older. This research setting was chosen due to the research team members’ previous experience and research in the community and collaboration with this specific LGA council. Participants were recruited via convenience sampling through community organisations and network contacts who disseminated recruitment material across Cardinia Shire including through social media. This included Cardinia Shire council and council networks, organisations and charities working with community members, health services and a food market social enterprise. Recruitment commenced in July 2021 and data collection concluded in October 2021. During this period people residing in metropolitan Melbourne, including Cardinia Shire, were subjected to two state Governments enforced COVID-19 lockdowns, 85 days in total. These lockdowns involved individuals staying home unless visiting essential retail within 5 km of their home (e.g. grocery stores, takeaway food), medical services or exercising outdoors before a 9 pm to 5am curfew and involved working or studying from home where possible. Therefore, online Zoom (Zoom version 5.11.1 [37]) or telephone interviews were the only viable options during this time. For completing both parts of the study, the participants received an AU$60 voucher to a local social enterprise fruit and vegetable market.

### Data collection

#### Part 1 quantitative survey

A link was included in the recruitment email or flyer to take participants to the Qualtrics XM^Ⓡ^ survey (Qualtrics Version July 2021, Provo, Utah [38]). Explanation of the study and consent form were embedded in the opening survey page. For those who were eligible and consented to take part in both parts of the research, a 15-min demographic, food access and food security survey was then completed. This survey had previously been implemented by Cardinia Shire council in 2021 to measure food security status across a larger representative sample of households. Demographic information included age, gender, country of birth, employment, income, education, and household structure. The survey also included questions related to food access such as the main grocery buyer for the household, stores they usually purchase groceries from, transport used, grocery shopping frequency and factors (cost, place of origin, quality/freshness, and appearance) that are important when purchasing food.

Additionally, to gather an understanding of the food security status and associated determinants, questions exploring money for food, barriers to purchasing food (e.g. too many other things to pay for and food costs too much) and coping strategies used (e.g. asked family or friends for help, accessed emergency food relief). To determine food security status over the last 12 months the USDA Household Food Security Survey Module—18 item (USDA-HFSSM) was implemented and scored according to the protocol [32]. The USDA-HFSSM is validated and consists of 10 questions for adults and 8 additional questions only for those who have children less than 18 years old in their household [32]. Participants were categorised into four food security status categories based on the number of affirmative responses to; ‘high food security’, ‘marginal food security’, ‘low food security’ and ‘very low food security’ [32]. As supported in the Canadian literature [39], for binary classification of food secure and food insecure we included people characterised as experiencing marginal food security as food insecure in addition to low and very low food security. This recognises that the experiences and outcomes of marginal food security are more consistent with food insecurity. Additionally due to the context of the time of the interviews during the COVID-19 pandemic, participants were asked how many of their food challenges experienced have been due to the pandemic.

#### Part 2 qualitative photovoice method

Photovoice is a participatory empowerment research method which allows participants to photograph their experiences with the aims to 1) enable people to document, through photographs, the strengths and their concerns with their community; 2) encourage discourse around important issues through discussion of the photographs with small or large groups, and; 3) reach policymakers in the area of concern [40]. The photovoice technique has been previously used by Chilton et al. [41] in the “Witnesses to Hunger” program, a research and advocacy project partnering with mothers and caregivers of young children who have experienced hunger and poverty. Photovoice is able to uncover the needs as well as the current assets of the community from the eyes of the community themselves, which can help to inform future decision making [40].

#### Photograph taking activity

Participants who completed the survey and consented to the photovoice activity were provided with detailed instructions by the researchers via a telephone call and emailed follow up written material with visual examples. Participants were asked to take photographs of anything they believed was important in relation to access to food for themselves and their household. This included, but was not limited to, grocery shopping, transport, food options, food retailers, storing food, preparing food and cooking food. Participants were given two weeks to take photographs using their own device if available (e.g. mobile phone camera or digital camera) before their scheduled interview. All participants took photographs using their mobile phone cameras and either emailed the photographs or shared through online private file sharing prior to the interview. Researchers then uploaded all photographs to be stored and accessed on LabArchives™ platform [42] in accordance with the approved ethical requirements.

#### Interviews

One-on-one photovoice interviews were conducted by authors SK and AM from August to October 2021. All interviews were conducted at a time suitable for both interviewer and participant via the videoconferencing program Zoom (version 5.11.1 [37]) or telephone. Interviews conducted via Zoom involved the interviewer sharing their computer screen, so participants and interviewer were able to look at the photographs together. Telephone interviews involved the interviewer and participant both having the photographs with them and the participant describing each photograph to make sure both were viewing the same photograph.

Interviews were semi-structured and explored the meaning behind the photograph by discussing photographs one-by-one. The average number of photographs taken by participants was 15 (range 0–45). Participants were asked to describe what the photograph was, what was happening in the photograph, what this means to them and meaning in relation to their access to food. Follow-up questions aimed to further clarify meaning of what was discussed by participants or to explore further areas of food access, such as what supports their food access, difficulties accessing food, aspects of their local food environment that need to change to support food access and any ideas for change. Concluding questions aimed to allow expansion and emphasis of any points participants had discussed throughout the interview. Due to personal circumstances one participant was not able to take photographs  and  completed an interview covering access to food, follow-up questions and concluding questions.

Interview duration was between 34 to 64 min. All interviews were audio recorded with participants consent and transcribed verbatim using a professional transcription service. Interviews were conducted by either of two authors (SK and AM) both with experience in qualitative interviewing. SK is a public health nutrition researcher and dietitian who is involved in work in the target community of this research and with previous experience in the photovoice method. AM is a public health nutritionist without previous work experience in the target community and a photovoice technique novice who received instruction prior to conducting interviews. The authors introduced themselves as researchers rather than nutritionist or dietitians to minimise participant potential of feeling judgment based on their food decisions and aimed to encourage discussion of other influencing factors. Field notes were taken throughout the interviews and used as a form of reflection throughout subsequent interviews through alterations to questioning style and in the analysis process to reflect on codes and themes. Authors came together throughout the study to reflect and discuss the interviews and to refine the interview process.

### Data analysis

Data from the survey were analysed using the statistical software package IBM^Ⓡ^ SPSS Statistics for Apple macOS version 28.0. Food security status was determined using the USDA-HFSSM scoring system [32]. Descriptive statistics, count and frequency are reported.

Qualitative analysis of the photovoice interviews was undertaken by the two authors who conducted the interviews (SK and AM) and involved inductive thematic analysis [43]. The analysis process involved familiarisation with the interview transcripts by listening to the audio recordings while reading the transcripts. Both authors then independently coded a subset of three interviews before coming together to triangulate and discuss their codes and assess inter-coder agreement. This discussion of initial open coding was used to develop an initial coding framework. Authors then each independently coded a subset of the remaining interviews using this coding framework. Throughout coding subsequent transcripts, this coding framework was iterated and further developed with authors coming together to discuss when iterations were suggested. Themes were developed through discussion between the two authors based on the prominent codes amongst the coding framework.

The contents of the photographs from participants were not coded separately to their corresponding interview excerpt. This was due to the intrinsic link between the photograph and the corresponding discussion by the participant and the need to take both data points together in context. The software program QSR NVivo (version 20 [44]) was used to manage and store interview data and for coding of the interviews as well as mapping sections of the interviews to the corresponding participant photograph.

## Results

### Part 1 quantitative survey results: demographics and food security status

There were 17 participants who completed the study. Twelve participants were categorised as experiencing food security and five participants experiencing food insecurity (‘Marginal food security’ *n* = 1, ‘Low food security’ *n* = 2 and ‘Very low food security’ *n* = 2).

Participants were primarily female (*n* = 14) and aged between 25–49 years (*n* = 10) (Table 1). Participants most commonly were from a household of a couple with children (*n* = 9), of which three were experiencing food insecurity and were homeowners with a mortgage (*n* = 10), of which three were experiencing food insecurity. Employment status and income varied with the most common work status for those experiencing food insecurity being part-time/casual paid work (*n* = 3) and household income between AU$0—AU$103,999 (*n* = 5).

**Table 1:** Participant demographic characteristics (*n* = 17) according to food security status

Characteristic	Categories	All participants n (%)	Food secure (*n* = 12)	Food insecure (*n* = 5)
**Age**	18–24 years	1 (5.9%)	0 (0.0%)	1 (20.0%)
	25–34 years	5 (29.4%)	2 (16.7%)	3 (60.0%)
	35–49 years	5 (29.4%)	5 (41.7%)	0 (0.0%)
	50–59 years	3 (17.6%)	2 (16.7%)	1 (20.0%)
	60–69 years	2 (11.8%)	2 (16.7%)	0 (0.0%)
	70–84 years	1 (5.9%)	1 (8.3%)	0 (0.0%)
**Gender**	Female	14 (82.4%)	10 (83.3%)	4 (80.0%)
	Male	3 (17.6%)	2 (16.7%)	1 (20.0%)
**Living arrangements**	Homeowner with a mortgage	10 (58.8%)	7 (58.3%)	3 (60.0%)
	Homeowner with no mortgage	3 (17.6%)	3 (25.0%)	0 (0.0%)
	Renting	3 (17.6%)	1 (8.3%)	2 (40.0%)
	Retirement village	1 (5.9%)	1 (8.3%)	0 (0.0%)
**Household type**	One person	2 (11.8%)	2 (16.7%)	0 (0.0%)
	Couple with no children	5 (29.4%)	4 (33.3%)	1 (20.0%)
	Couple with children	9 (52.9%)	6 (50.0%)	3 (60.0%)
	Group household	1 (5.9%)	0 (0.0%)	1 (20.0%)
**Highest level of education completed**	Year 7–9 secondary school	1 (5.9%)	0 (0.0%)	1 (20.0%)
	Year 10–11 secondary school	2 (11.8%)	2 (16.7%)	0 (0.0%)
	Year 12 secondary school	2 (11.8%)	2 (16.7%)	0 (0.0%)
	Certificate (trade or business)	1 (5.9%)	1 (8.3%)	0 (0.0%)
	Diploma or TAFE^a^	1 (5.9%)	1 (8.3%)	0 (0.0%)
	Bachelor degree	6 (35.3%)	3 (25.0%)	3 (60.0%)
	Graduate diploma or graduate certificate	2 (11.8%)	2 (16.7%)	0 (0.0%)
	Postgraduate degree	1 (5.9%)	1 (8.3%)	0 (0.0%)
	Prefer not to say	1 (5.9%)	0 (0.0%)	1 (20.0%)
**Employment status**	Full-time paid work	2 (11.8%)	2 (16.7%)	0 (0.0%)
	Part-time paid work	4 (23.5%)	2 (16.7%)	2 (40.0%)
	Casual paid work	1 (5.9%)	0 (0.0%)	1 (20.0%)
	Self-employed	1 (5.9%)	1 (8.3%)	0 (0.0%)
	Volunteering	1 (5.9%)	1 (8.3%)	0 (0.0%)
	Home duties	4 (23.5%)	3 (25.0%)	1 (20.0%)
	Carer	1 (5.9%)	0 (0.0%)	1 (20.0%)
	Retired	3 (17.6%)	3 (25.0%)	0 (0.0%)
**Annual household income**	AU$0—AU$25,999	3 (17.6%)	1 (8.3%)	2 (40.0%)
	AU$26,000—AU$51,999	4 (23.5%)	3 (25.0%)	1 (20.0%)
	AU$52,000—AU$103,999	7 (41.2%)	5 (41.7%)	2 (40.0%)
	AU$104,000—AU$207,999	3 (17.6%)	3 (25.0%)	0 (0.0%)
**Country of birth**	Australia	15 (88.2%)	11 (91.6%)	4 (80.0%)
	United Kingdom	2 (11.8%)	1 (8.3%)	1 (20.0%)

^a^TAFE Technical and Further Education

In relation to food acquisition (Table 2), *n* = 13 participants were the main grocery buyer for their household. Participants primarily shopped at a store 1–5 kms from their home within Cardinia Shire (*n* = 8). Food insecure participants mostly used a car to access groceries (*n* = 4) while one participant used public transport. Participants mainly used supermarkets (*n* = 15) and specialty stores (e.g. greengrocer or wholesale market) (*n* = 12) to purchase fruit and vegetables. On average participants considered quality and freshness to be the most important factors when shopping for fruit and vegetables, with those experiencing food insecurity placing a higher importance on cost. The most commonly used coping strategies when there was not enough food included shopping at a range of food outlets for food discounts (*n* = 5) and using emergency food relief or food banks (*n* = 3). These coping strategies were primarily used by those experiencing food insecurity but not exclusively.

**Table 2:** Food security status and food access characteristics according to food security status

Question	Categories	All participants (*n* = 17)	Food secure (*n* = 12)	Food insecure (*n* = 5)
Are you the main grocery buyer for your household?^a^	Yes	13 (76.5%)	9 (75.0%)	4 (80.0%)
	No, but I do shop for groceries sometimes	4 (23.5%)	3 (25.0%)	1 (20.0%)
Where do you mainly do your grocery shopping?^a^	A store less than 1 km from home	4 (23.5%)	3 (25.0%)	1 (20.0%)
	A store 1 km to 5 km from home	8 (47.1%)	6 (50.0%)	2 (40.0%)
	A store more than 5 km from home	2 (11.8%)	1 (8.3%)	1 (20.0%)
	Home delivery	2 (11.8%)	1 (8.3%)	1 (20.0%)
	Local farms and growers	1 (5.9%)	1 (8.3%)	0 (0.0%)
What is your main form of transport?^a^	Car	15 (88.2%)	11 (91.7%)	4 (80.0%)
	Walking	1 (5.9%)	1 (8.3%)	0 (0.0%)
	Public transport	1 (5.9%)	0 (0.0%)	1 (20.0%)
How often do you buy fresh fruit and vegetables from a supermarket?^a^	Never	2 (11.8%)	1 (8.3%)	1 (20.0%)
	Once a week	8 (47.1%)	6 (50.0%)	2 (40.0%)
	2–3 times a week	6 (35.3%)	4 (33.3%)	2 (40.0%)
	Most days	1 (5.9%)	1 (8.3%)	0 (0.0%)
How often do you buy fresh fruit and vegetables from a specialty store (e.g green grocer or specialty store)^a^	Never	5 (29.4%)	3 (25.0%)	2 (40.0%)
	Once a month or less	6 (35.3%)	4 (33.3%)	2 (40.0%)
	2–3 times a month	4 (23.5%)	3 (25.0%)	1 (20.0%)
	Once a week	2 (11.8%)	2 (16.7%)	0 (0.0%)
How often do you buy fresh fruit and vegetables from a farm or farmer (e.g. farmers market, box scheme, farmgate)^a^	Never	8 (47.1%)	6 (50.0%)	2 (40.0%)
	Once a month or less	7 (41.2%)	5 (41.7%)	2 (40.0%)
	Once a week	2 (11.8%)	1 (8.3%)	1 (20.0%)
When shopping for fresh fruit or vegetables how important are the following factors^b^	Cost	3.94 (0.97)	3.75 (0.97)	4.4 (0.89)
	Place of origin	4.12 (0.86)	4 (0.95)	4.4 (0.55)
	Quality/freshness	4.88 (0.33)	4.83 (0.39)	5 (0)
	Appearance	3.71 (1.05)	3.58 (1.16)	4 (0.71)
To what extent have food challenges experienced been due to changes in circumstances of the COVID-19 pandemic?^a^	All food challenges in the last year due to impacts COVID-9	2 (11.8%)	0 (0.0%)	2 (40.0%)
	Most food challenges in the last year due to impacts of COVID-19	1 (5.9%)	0 (0.0%)	1 (20.0%)
	Some food challenges in the last year due to impacts of COVID-19	3 (17.6%)	1 (8.3%)	2 (40.0%)
	Not experienced any food challenges in the last year	11 (64.7%)	11 (91.7%)	0 (0.0%)
In the last 12 months what have you done to cope with not having access to enough food?^a^	Visited family and/or friends specifically to eat at meal time and or snacks	2 (11.8%)	0 (0.0%)	2 (40.0%)
	Asked family and/or friends for food	1 (5.9%)	0 (0.0%)	1 (20.0%)
	Asked family or friends for money for food	2 (11.8%)	0 (0.0%)	2 (40.0%)
	Shopped at a number of food outlets for food specials/discounts	5 (29.4%)	1 (8.3%)	4 (80.0%)
	Used emergency food relief or food banks to get food items or vouchers for food	3 (17.6%)	0 (0.0%)	3 (60.0%)
	Accessed or received meals for no to low cost from organisations	2 (11.8%)	0 (0.0%)	2 (40.0%)
	Accessed or received financial assistance from organisations	2 (11.8%)	0 (0.0%)	s (40.0%)

^a^n (%)

^b^Mean (standard deviation)

### Part 2: qualitative photovoice interview results

**Fig. 1:**
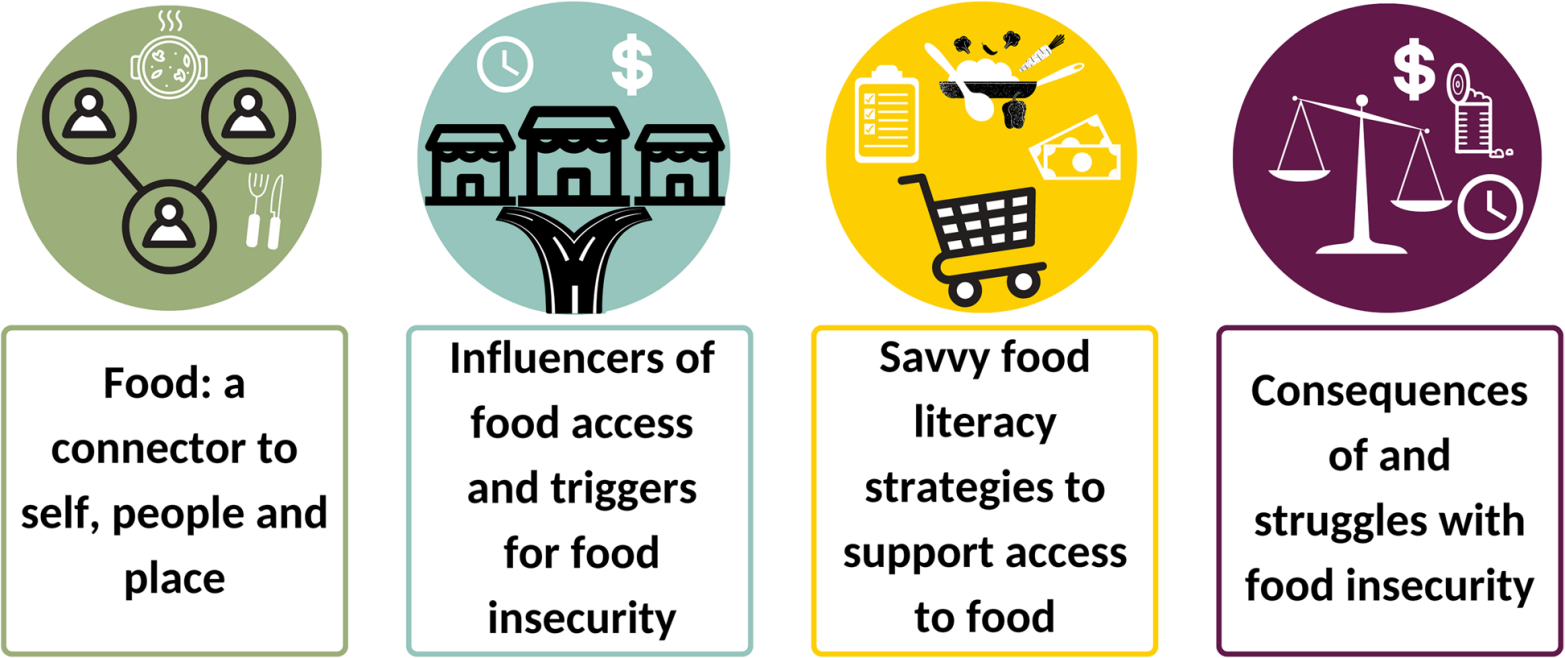
Themes of photovoice interviews with community members

Through thematic analysis of the photovoice interviews four themes with six sub-themes and suggestions for change were developed (Fig. 1). The themes and sub-themes are presented below along with photographs that prompted some of the corresponding participant quotes.

### Theme 1: Food: a connector to self, people and place

Food was viewed as an important connector to self, friends, family, community and land. Participants discussed the way food can bring people together and a community can be built through: sharing food, helping others out with food, connection with local food producers and community initiatives such as community gardens. It is important to note that while most community members discussed the ability for food to nurture social and emotional health, this was not necessarily the case for those experiencing food insecurity.

#### Sub-theme 1a: Food nurtures social and emotional health

Food was seen as an important aspect of life that went beyond physical nourishment. Most participants shared photos and described how food was special to them, used in times of celebration and supported their emotional and social health.

“Food is a part of life, it’s not just for eating because you’re hungry. It’s always part of the celebrations as well.” (Kelly, experiencing food security)

Participants described food as a connector to their social circle and sometimes this connection extended to the wider community. Some participants highlighted that shopping in a local environment and supporting local businesses was a rewarding experience, garnered a sense of connection to the food and the people producing and selling it, and was seen as an important part of their routine.

“I love walking down the street and buying the food from my local sort of strip shopping. I love that atmosphere, that feeling. This is another highlight.” (Mary, experiencing food security)

For people who were experiencing food insecurity, this positive connection to food was often challenged. Food could be a source of stress or anxiety rather than a positive influence on emotional health. A positive connection to food was sometimes hindered by feelings of uncertainty and the need to make sacrifices, sometimes missing out on the enjoyment of food.

“I think it puts a little bit more stress on if things go bad or if there’s those things that you don’t account for, you’re probably more stressed about that. I think, though, sometimes, when it comes down to it, you’ve gotta make decisions at the time when you’ve gotta make them and then you’ve just gotta sit out the rest of the time.” (Michelle, experiencing food insecurity)

#### Sub-theme 1b: Building a community around food

As well as food supporting personal social connections, food was also discussed as a way of creating or enhancing a sense of community. Participation in community projects, such as community gardens, not only connected participants with people they may not have known otherwise, but also allowed for a greater understanding and appreciation of where food came from.

“I think building a community around food or food availability is important, like the community gardens. I think they’re a really important resource that are underutilised at the moment.” (James, experiencing food security)

For some participants who were food secure, living in smaller townships had a positive influence on their experience accessing food. They were able to visit local food retailers close to them and often knew the people working there, which enhanced a feeling of community connection. They were able to buy quality produce which was a priority while knowing they are supporting both community retailers and local growers.

**Fig. 2:**
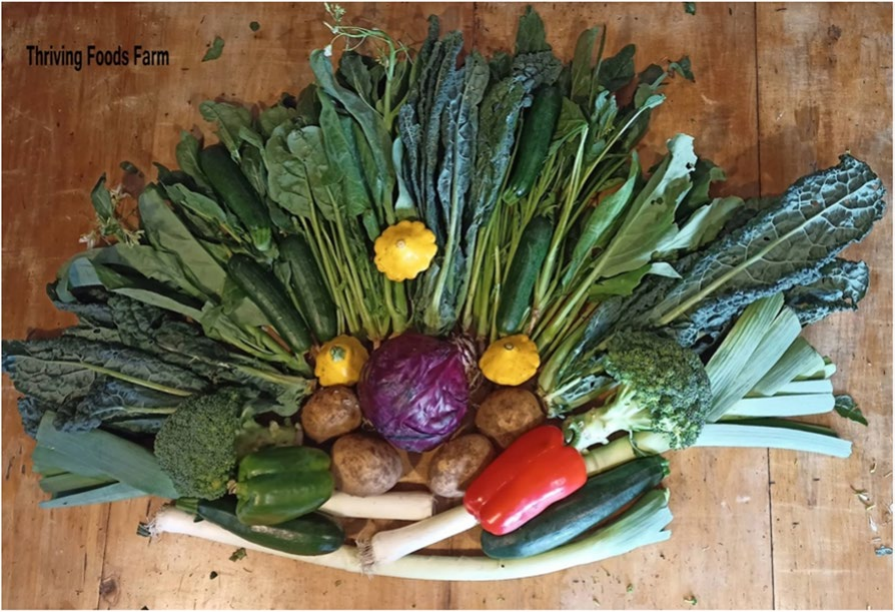
Participant photograph ‘Building a community around food’

“You’re supporting a small business, and local. It’s small, that’s the key, that there’s lots of small farmers. I think when you keep it small, not trying to get so big, you can really dedicate the quality, or the farmer can dedicate their quality and time to growing a really good crop.” (Mary, experiencing food security referring to Fig. 2)

Communities came together to support others. Participants described providing their neighbours with fresh produce they had grown or exchanging produce with people from across the wider community. Other participants described providing food to food relief organisations or to help people experiencing food insecurity, strengthening their connection with their community.

**Fig. 3:**
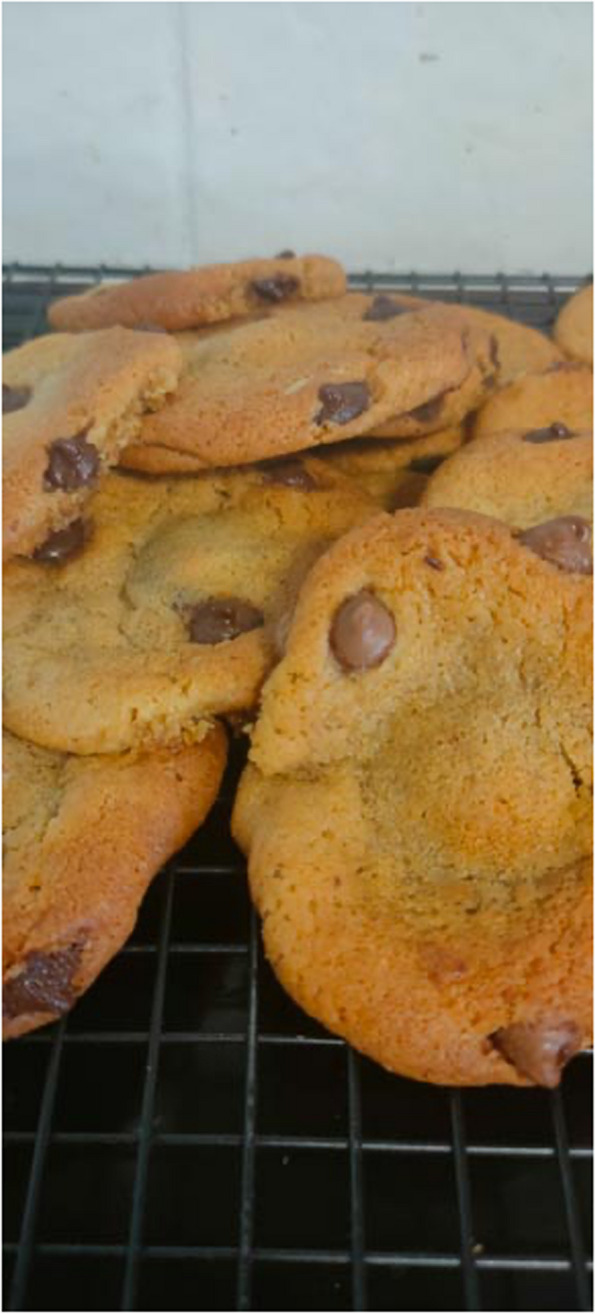
Participant photograph ‘Building a community around food’

“Because chemistry is a hard subject, every week I used to make cookies for the chemistry students. I can’t do that because we’re not allowed to share food now. I know—not the staff particularly—but I know the kids at that school, some kids have food access issues. I think at some point that cookie might have been the first thing that they ate for the day.” (Kelly, experiencing food security referring to Fig. 3)

### Theme 2: Influencers of food access and triggers for food insecurity

There were many influencers that played an important role in the ease of access to quality, nutritious food. People described supportive influencers to food access such as having: a variety of food retailers close to them, transport to do grocery shopping and money available. For some people these aspects were triggers for food insecurity including competing and unexpected personal and household costs, cost of food, time taken to shop and cook and having the physical hardware to access and cook food.

#### Sub-theme 2a: External drivers

The local food environment was a widely discussed driver for how and why participants accessed the food that they did. People discussed the layout of their town, which food retailers were easy to get to, as well as which areas had multiple food retailers conveniently located close together. When people used independent food retailers such as butchers or greengrocers, they primarily accessed those that were near other grocery retailers. Factors such as distance away from home or work, the amount of traffic, as well as ease of parking were important in grocery outlet decision making. Some participants, particularly those in smaller towns, described using independent local food outlets rather than relying on larger chain supermarkets.

“Having things in local shops to make it a lot easier, having the space and the ability to grow vegetables and have my chickens and that kind of thing. Having that little bit more income to be able to purchase the quality of ingredients and not have to worry so much about that importance.” (James, experiencing food security)

Consistent with the quantitative (Part 1) results, most participants described predominantly using major supermarket chains for the bulk of their grocery shopping. The reasons for this mainly revolved around convenience, lack of other retail options, price, and familiarity. Some participants felt they had restricted choice in where they could obtain their groceries, with limited variety within close proximity or other food retailers being perceived as out of their budget. There was also limited choice of food for those relying on food relief. Most participants relied on cars as their main form of transportation, with some describing a lack of alternatives. The local accessible public transportation was thought to be inadequate or too difficult to use when grocery shopping as they did not go near food shops or were at infrequent times. One participant who relied on a bicycle for transportation described how their town was not well set up or safe for those who used bicycles.

**Fig. 4:**
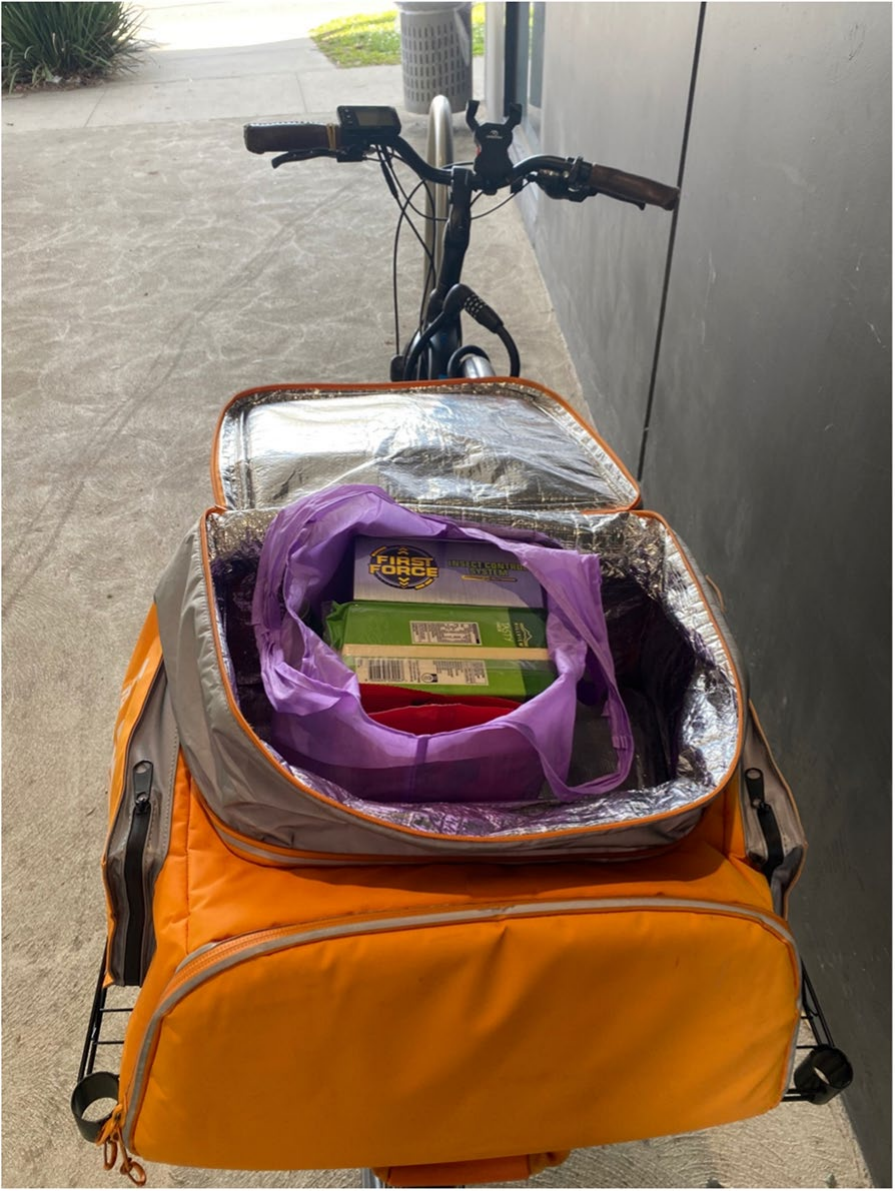
Participant photograph ‘External drivers’

“It [riding a bike] is not always the safest, I wouldn't say, the safest transport that you can get around. Yeah. When I was living in the city, absolutely. The City of Melbourne it's amazing to ride bicycles. They have really good bike lanes and everything. Around here not as much.” (Lucas, experiencing food insecurity referring to Fig. 4)

Other important external drivers for food access were the quality and variety of produce and the cost of food variations, with quality being classified as highly important in the Part 1 survey. Produce from  certain stores were described as of higher quality, such as fresh food markets and green grocers. However, many also felt it was a necessity to find a balance between cost and quality, as the high quality they sought often came at a price.

#### Sub-theme 2b: Personal and household influencers

Personal financial circumstances were viewed by many participants as key in their food purchase decision making. The discussion around money varied between participants, with some describing feeling lucky for being able to afford what they need and others being conscious of their money and having to tightly manage their finances.

“I know- I’m better positioned as far as access to food and money to purchase food, I’m very much well aware there are other people who aren’t.”( Karen, experiencing food security)

With many competing costs including rent or mortgage payments, other bills, medical expenses and petrol, money to pay for food was restricted for some participants. Changing personal circumstances and new priorities such as changing jobs and expanding families, also greatly affected the amount of money people had available to purchase food.

“At the moment, with the financial constraints, I do prefer to buy home brand because it’s cheaper. Before having my bub, I probably was spending a bit more time looking into the brands. Considering the price, but also preferring to go with the Aussie brands. I guess, with that, it does come with a slightly higher price tag, bit harder to do that now.” (Kimberly, experiencing food insecurity)

Participants had differing personal beliefs and priorities when it came to food they purchased, including health, ethics, and sustainability. Many participants described buying local Australian produce as a priority while some others also placed importance on knowing where the location food was from and the ethical treatment of animals.

“There’s probably—there’s a lot of reasons, but one part of it is sustainability. I just, yeah, with transport and all of the other factors that go into actually producing food. I’ve kind of developed an interest over the last probably 15 years in just trying to reduce the impacts as much as possible in that regard. Making anything from scratch, or particularly from our garden, is with minimal materials and certainly no waste, is very important to me.” (Mark, experiencing food security)

To be able to buy and use the food efficiently it was important to participants to have the appropriate physical hardware. Having enough storage space and cooking utilities that properly functioned determined what food people purchased, the frequency of purchasing food, as well as meal types. Car access allowed for participants to make larger trips to the grocery store to purchase food and therefore required less frequent shopping trips, which saved on both time and sometimes money when buying in bulk.

**Fig. 5:**
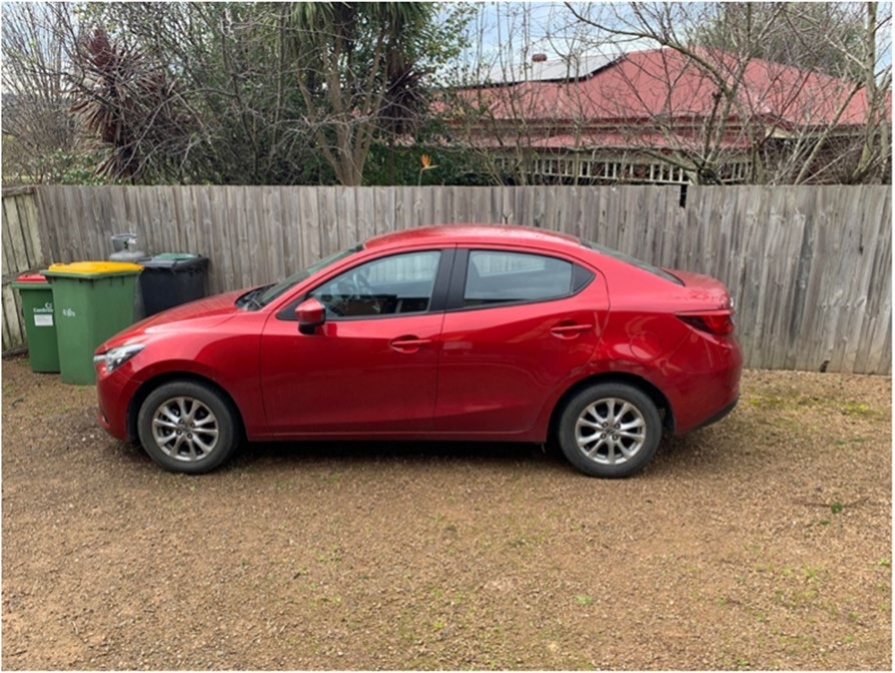
Participant photograph ‘Personal and household influencers’

“…transport to the shops, and it's always car, like I was saying. Because we're shopping for so many people, it would be hard even if the supermarket was around the corner. Unless I could push the trolley home, it would be quite hard to do a big shop and bring it home.” (Sarah, experiencing food insecurity referring to Fig. 5)

Medical conditions and poor physical or mental health not only impacted how participants could obtain food, including the ability to get to the shops and to prepare and cook food, but also income and therefore money available for food.

“Unfortunately, I don’t get anything (money) from Centrelink, because my disability isn’t something they can acknowledge. I don’t work like I used to, so it’s very hard for me to – we’re just sort of trying to live on one wage, especially, even more with COVID. It’s a matter of just buying the minimum that I can, and my kids are older; but it doesn’t matter, they still live at home. If I go without, I go without. I haven’t had breakfast or anything today.” (Susan, experiencing food insecurity)

### Theme 3: Savvy food literacy strategies to support access to food

People described using many different strategies to access the food they need in the best way possible. The strategies described comprised different aspects of food literacy, which can be defined as the inter-related skills, knowledge and behaviours that are required for a person to be able to plan, access, prepare and cook the food that they need [45].

Irrespective of food security status, a key aspect of food literacy discussed was planning and shopping strategies which involved different knowledge sets and skills. Some participants mentioned planning when it is best to go grocery shopping, where the most convenient places to obtain food were and what they need to buy, with some people mentioning shopping lists or planning meals for the week. Additionally, participants mentioned shopping for discounted food, shopping across multiple retailers for discounts and choosing ingredients that were versatile and could be used across a range of meals. Importantly, cooking skills were also mentioned as a key strategy to food access. Having the knowledge of how to use different ingredients to make meals was important to assist with utilising seasonal or discounted ingredients they may have.

**Fig. 6:**
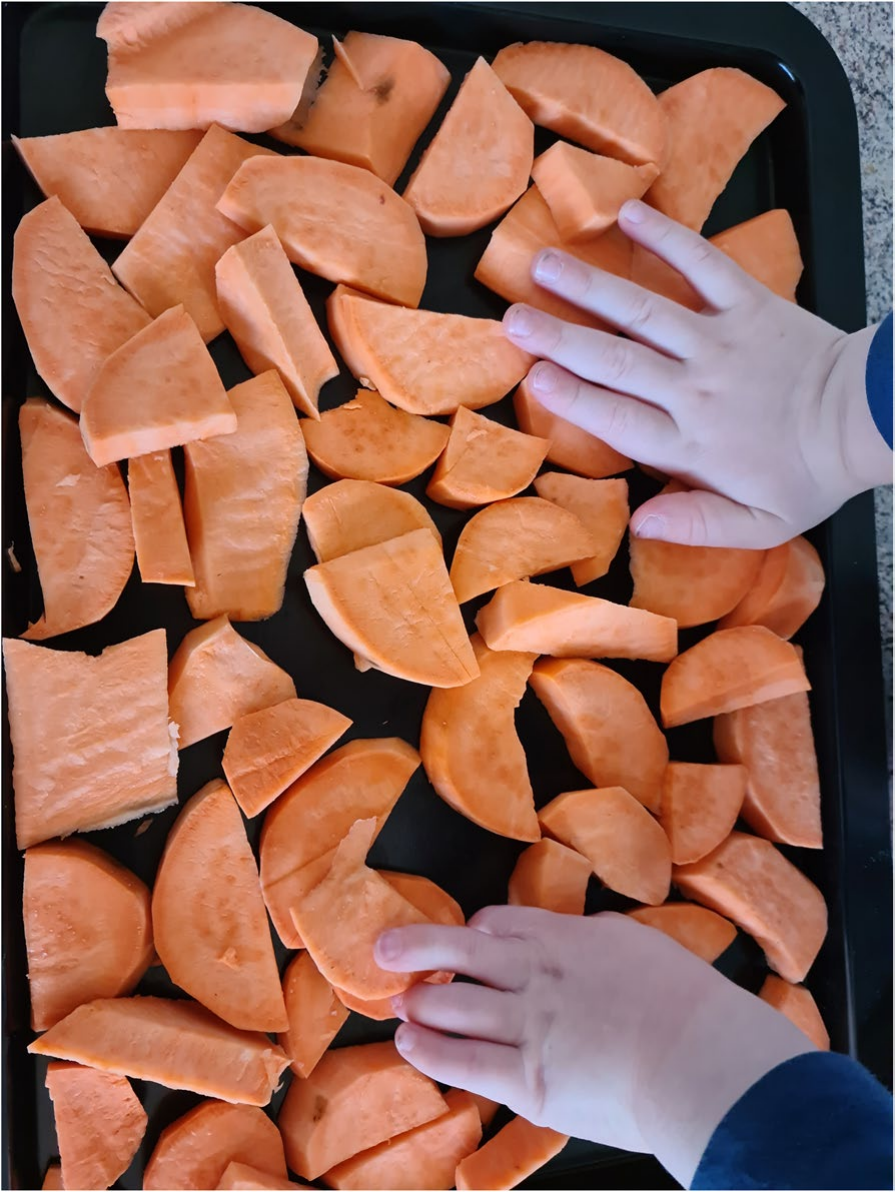
Participant photograph ‘Savvy food literacy strategies to support access to food’

“It’s cooking what we have at the time. We didn’t have any potatoes, we didn’t have pumpkin, we had sweet potatoes. So, instead of a tray of mixed vegetables, it was a tray of sweet potato, which he loves, I love. So it certainly was eaten, but, yeah, it’s about cooking what we have in abundance.” (Michelle, experiencing food insecurity referring to Fig. 6)

Together with the food they obtained from stores, many participants mentioned growing some of their own food. The extent of how much and what types of home grown produce they grew varied amongst participants, but all still relied on stores to get some or most of their food due to the small scale of home-grown produce. Growing produce was a hobby for some and others had just begun as an activity during COVID-19 lockdowns, especially as an alternative to fresh produce from farmers markets which were not open during those times.

**Fig. 7:**
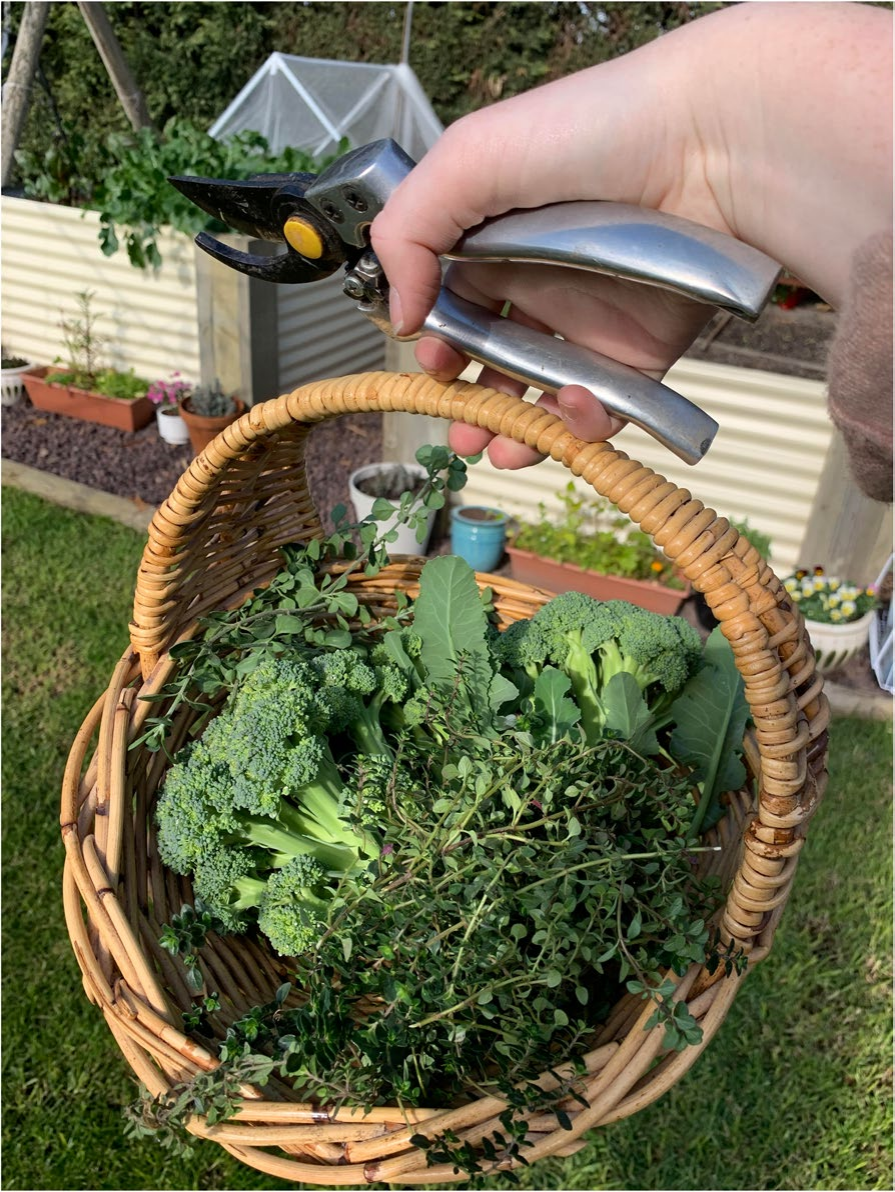
Participant photograph ‘Savvy food literacy strategies to support access to food’

“We’ve been growing a lot more food to try to limit what we’re getting from the supermarket, both from a plastic perspective and then how far our food is coming from a mileage perspective.” (Sarah, experiencing food insecurity referring to Fig. 7)

Participants described different storage and food organisation strategies for storing food and keeping it fresh for longer, which were reliant on the physical hardware they had for storage, for example, additional or large freezers. These included strategies like transferring food out of packets into airtight containers and freezing and rotating food.

“I have lots of containers for the fridge that I got from the op-shop. If you just put in a nice, little airtight container in the fridge, things last. Generally, I use them up. You get a lot more use out of it. You don’t throw it out” (Janet, experiencing food security)

These strategies helped participants use up food bought in bulk, save money and reduce food waste. However, in order to bulk buy and have the level of storage organisation was coupled with the need for financial resources and subsequently was not achievable for all participants.

Alongside mentioning their own food literacy knowledge and skills, a few participants with children discussed passing along this food literacy to their children. They wanted their children to know where food comes from and how to prepare it, in the hopes that their children would adopt healthy habits as they grew up.

“I think now having a little one that's in the backyard a lot more, I've always said, "Oh, I wanna teach him how to garden and where food comes from." Not that we could ever do it to a large scale, but I think it's important to see where food comes from and healthy food, I suppose.” (Laura, experiencing food security)

### Theme 4: Consequences of and struggles with food insecurity

Participants who were experiencing food insecurity described their experiences and consequences they faced accessing food. Regardless of food insecurity severity, many participants described shared struggles. There were many competing factors that affected the ability to access the food they need including money, competing bills, health, time and cost of food. For some participants, utilising food relief services was an important, and at times the only way to access food. Despite a sense of gratitude for these services a range of issues were highlighted.

#### Sub-theme 4a: Diverse but overlapping consequences

Accessing food while experiencing food insecurity was a balancing act. Participants described having to decide how much of their money they would have to use for essentials such as bills, while also trying to save enough to buy food. The amount of money people had to spend on food varied from week to week, with some weeks being harder to put food on the table. Tight budgeting and planning strategies were used, but despite this sacrifices and trade-offs were made to pay bills where food was often being designated to what money was ‘left over’.

**Fig. 8:**
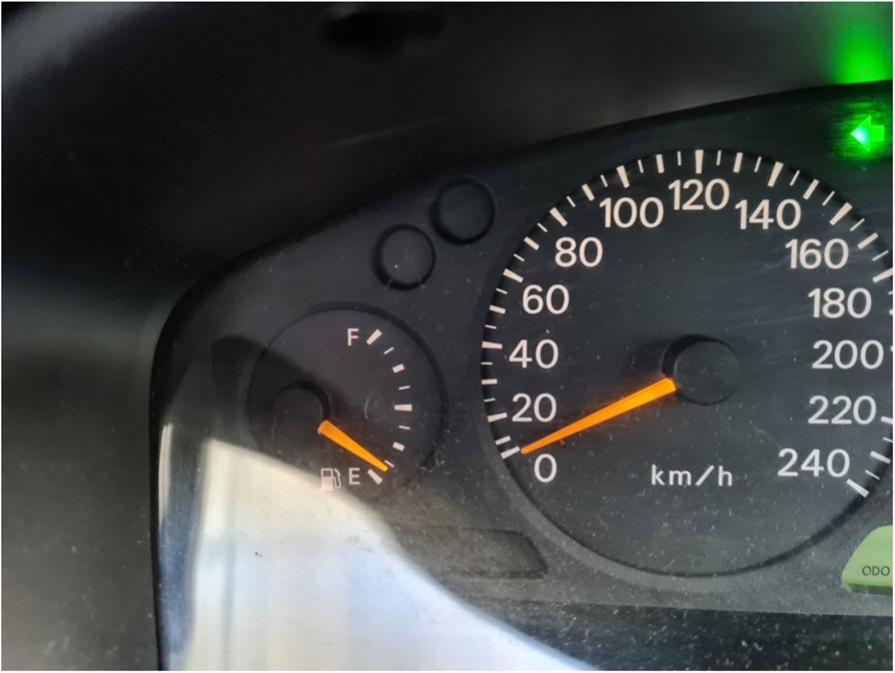
Participant photograph ‘Diverse but overlapping consequences’

“That’s about deciding- when you’ve still got the best part of a week till you have any more income – you’re nearly empty on your fuel and you’ve gotta decide whether you put your $20 in the car or put that towards food.” (Michelle, experiencing food insecurity referring to Fig. 8)

Trade-offs were common with the types of food participants could afford, choosing home brands and discounted or clearance products. This meant they were not always able to get food they may want or need as the discounts available, and their budgets, varied from week to week. Despite the participants experiencing food insecurity rating food quality as highly important (Part 1), there was often a trade-off on quality as discounted foods often had short shelf life and poorer quality.

“I always stop there [clearance section at supermarket] and look for things. I don't mind if they're on clearance. Sometimes they're a bit—the package is a bit damaged and stuff, but I just decided to put that picture on because if I can get something from over there, it always helps. It's been hard. I find a lot of good things on clearance over this last year, year and a half.” (Lucas, experiencing food insecurity)

Participants with children kept their children's interests as their top priority. It was of importance to protect their children or children in their community and allow them to have the food they want and need as much as possible.

“There’s a lot of really struggling kids. Lots of the kids I’ve heard are going to school without breakfast. Kids need to eat. How can they concentrate on their schoolwork when they’re hungry?” (Kelly, experiencing food security)

#### Sub-theme 4b: The 4 T’s to accessing food relief—Trial, tribulation, trade off and thanks

When money was scarce participants relied on food relief to access the food they needed. For some, this was their main source of food, with some participants accessing food relief for the first time during the COVID-19 pandemic and others relying more heavily on food relief during the pandemic than usual.

**Fig. 9:**
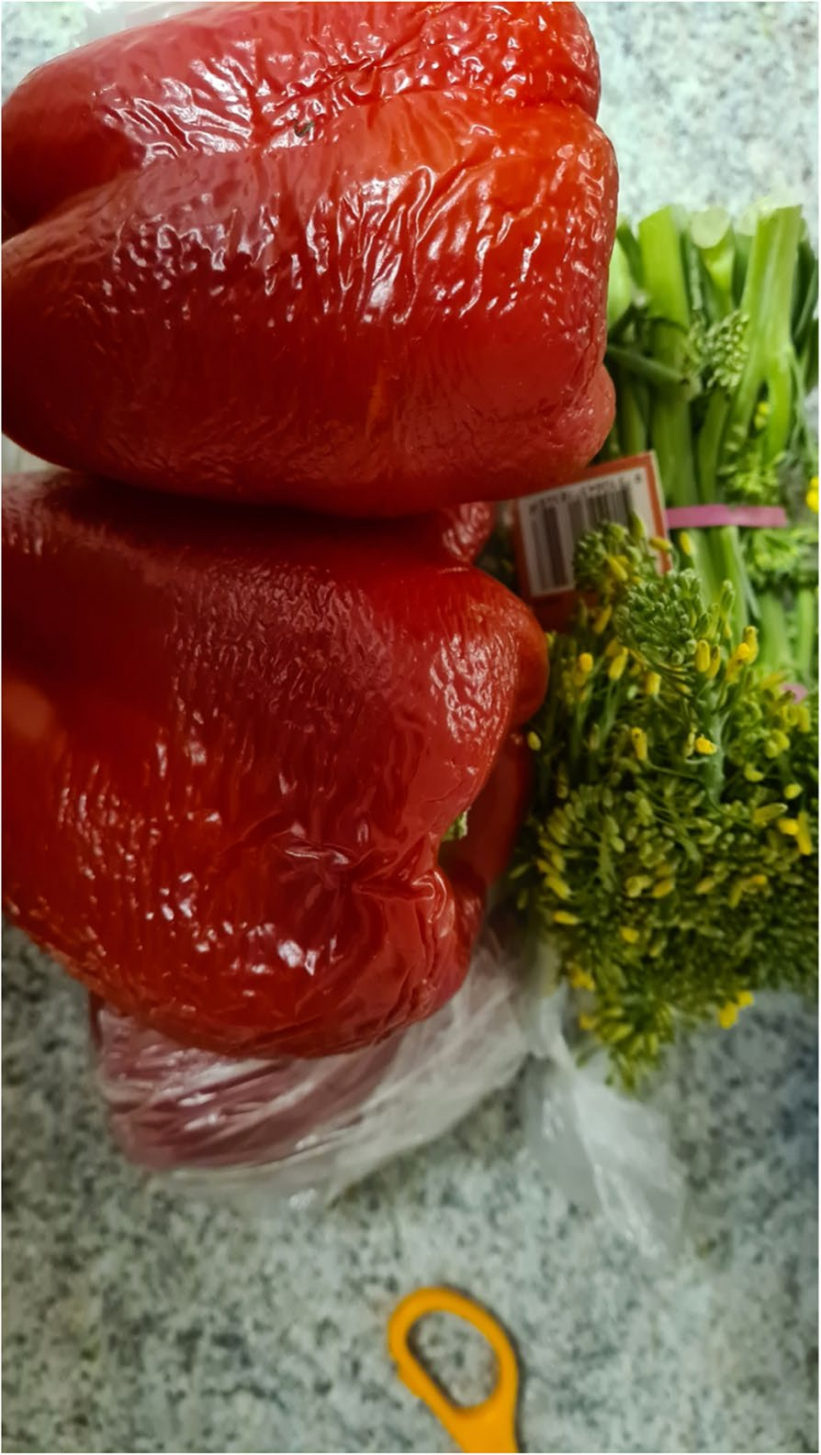
Participant photograph’The 4 T’s to accessing food relief—Trial, tribulation, trade off and thanks’

“We've been—this year, mainly, relying on some food support and that sort of thing, which is really great in the community and very, very helpful but I think that one thing that you've gotta take with that is the fact that you don't necessarily get—especially talking about vegetables—fruit and vegetables and meat. You don't have the luxury of getting necessarily fresh.” (Michelle, experiencing food insecurity referring to Fig. 9)

While participants expressed gratitude for the food available through food relief services there was a sense of lack of choice. This was related to a lack of options of where they could access food relief, as well as a lack of choice for how much and the types of food they could access. Some food relief services had limits on the number of items you could take from each section, such as fruit, vegetables, meat, and grain-based foods. It was noted that some people accessing food relief would take more food than others and subsequently participants described needing to get to certain food relief services at a certain time to be able to get the food they needed. During the stricter COVID-19 lockdowns, food choice was removed entirely when the food was pre-sorted and packaged for food relief recipients. Despite the lack of choice, participants made use of what they could get using their food literacy skills.

“So, ………we would like to use fresh milk, all the time, [laughter], my son loves milk, I use milk for a lot of things, cooking and that sort of thing and the skim milk powder is one thing we got through, I think it was [Food relief organisation]. I took a photo showing you don’t always use what you want, but you’ve gotta use what you have as well. It might not be the same, but it does serve the same purpose.” (Michelle, experiencing food insecurity)

The sub-par quality of the food, particularly fresh produce, provided by food relief organisations was mentioned by all participants who accessed food relief. Participants described receiving vegetables which were already wilting (see Figure 9), to produce that was close to expiration. Additionally, a lot of the food on offer was highly processed with less fresh produce. These types of foods did not always fit with the participants desire to eat healthily or with their dietary requirements. Despite the lack of quality and choice, participants did not always feel they had options outside of food relief so they took what they could get.

“I think since we don’t have other sources of food to access to, it’s hard to have a balanced and healthier diet. You just eat what you get and that’s it. You cannot really complain.” (Lucas, experiencing food insecurity)

It is important to note that participants accessing food relief highlighted some service structural barriers that impacted their dignity. Amongst these was the lack of choice described earlier, which prevented them from being able to access the food they need in a dignified way. Additionally, some food relief organisations required recipients to prove eligibility to access the food, such as by providing a health care card. For one participant, this brought a sense of shame, with them not wanting to have to prove in front of others that they were struggling and required food relief. There was also a sense of judgement from others when accessing food relief, which affected when, where and how they accessed these food relief organisations.

“They watched me, and I’m like “Is there something wrong?” “I’m watching how many you take.” That was just – I just left the stuff, said, “Don’t want it. Thanks.” I understand they’ve got to watch people, so that people – like you see, five bags of the one thing, but then the next person I’ll see do it, and they don’t get told anything.” (Susan, experiencing food insecurity)

### Future directions: Suggested future strategies for change

Through their lived experience in their local settings in Cardinia Shire, participants had a range of ideas of what would enhance and support access to food in a way that they need. There was an emphasis on affordable and readily available access to locally produced food, from farmers or community gardens. Participants discussed the importance to them of understanding where their food comes from and supporting locally grown where possible. Suggestions included more independent fresh produce stores, encouraging local supermarkets to stock more local produce, local farm produce boxes and food stalls, such as at community markets. Participants highlighted the need for more affordable locally grown food.

“Sometimes also I’ve noticed the stuff produced overseas can be a bit cheaper. I feel like that’s also something that needs to be changed. Why not make that more expensive so that we are more inclined to support our own companies and keep production and that here?” (Kimberly, experiencing food insecurity)

Social enterprise food markets were seen as desirable not only for supporting local food access, but for its affordability and quality, which was of key importance. Some participants highlighted they were not always available to go to the social enterprise market with limited opening hours and the location not being readily accessible, especially by public transport.

Access to more community gardens or public growing spaces was highlighted by some participants as a way to encourage growing produce and community connectedness.

“The retirement facilities grounds management-built planter boxes free of charge and so she (my neighbour) growing spring onions. She is more than happy for me to come and pick one if I need it. Sometimes she has herbs. I have a small pot of herbs – so we swap things as we need – we have an understanding that its ok to go and pick from each other’s small produce area.” (Judy, experiencing food security)

Community gardens were not widely available in Cardinia Shire, but there was interest by some participants who may not have room or capacity to grow produce at their own home. Alternative suggestions to community gardens included nature strip gardens, where space that is not usually utilised is able to be used to grow produce.

In conjunction with the food literacy skills of gardening, some food secure participants suggested more opportunities to learn about shopping on a budget and cooking. Cooking classes were thought to be useful to provide knowledge on how to utilise certain ingredients such as those they may receive as part of food relief or seasonal discounted produce.

“I think people could probably learn ways to access higher quality, healthier food at a much cheaper cost than they are currently paying. I think that comes down to education to do with how things are produced, how things are transported, but also how to prepare food.” (Mark, experiencing food security)

There was a heavy reliance on cars as a main form of transport amongst most participants and the community. Therefore, some participants discussed the need for other transport options to be more available and spanning across larger areas of the community. There was a desire for more accessible public transport and safer bike lanes to encourage active transport.

“It’s just hard to get out onto the highway(to get to the shops). I’ve gotta cross over the highway……, it’s got the most disgusting intersection. It’s dangerous, and I’m an older driver, so I won’t go that way. Then I have to go up all these little side streets to get back to my home. That’s why I prefer to shop at Officer. They’ve gotta be better planning for public transport”(Janet, experiencing food security)

Participants who accessed food relief highlighted the desire for more, easily accessible information about where they can access different food relief services. Improvements to the delivery of food relief services included being grounded in respect, fairness, privacy, and dignity. Participants had the desire for systems to focus on equal and fair treatment that were not judgemental, particularly when it came to monitoring how much food people picked up.

“I suppose I would like to go to a place where you’re not also got people staring at you— where you can go in one by one, and be in a room, and go grab some stuff, and then you’re able to go out where— because I don’t like going, when I go to these places, I go early so I don’t have to have other people looking at me and I think they are judging me.” (Susan, experiencing food insecurity)

To assist with food security amongst children, there was the suggestion that school lunch options could be provided in schools. Currently there were children who were going to school hungry, and the addition of a school lunch scheme would mean a reliable source of food for these children.

## Discussion

This mixed methods photovoice study describes factors influencing food access within an individual, household, and local community setting. Our results highlight structural issues influencing how food is accessed within the community, such as access to local food, transportation and variety and affordability of food retailers. Additionally, participants highlighted many personal and household factors which influenced what food means to them, what food they could afford and their knowledge and skills around food. These factors that influence food access were also triggers for food insecurity, with adequate and sustainable income, time and cost of utilities playing a role in the food security status of participants. The discussion by participants highlighted all dimensions within the definition of food security [2, 3] including; food availability across their local setting; both physical and monetary access to food; how they utilise the food they access; the stability of supply and cost of food from food retailers; sustainability of food including local produce and food miles; and their individual agency to choose what they eat and influence their local food system.

When accessing food in their local setting participants identified a range of limiting factors, including physical access through location of retailers, car access through roads and parking as well as public transport options. Previous research in Melbourne, Australia [46] found in urban growth LGAs, few households were close to a supermarket and public transport options were also limited and suggested the importance of local urban planning policy within areas to reduce health inequities. Physical access to supermarkets has been associated with higher fruit and vegetable intake regardless of car access, however this was dependent on close physical proximity to the supermarkets, highlighting the importance of food access in city planning [47]. Food was seen as important for social and emotional health and connection to others. The benefits of food that extend beyond the nutrients they contain is known, with particular importance on eating with others, food for special occasions and creating and enhancing social bonds [48]. Community gardening was described by some participants as a social connection  mechanism, while also highlighting the need for more opportunities for and awareness of community gardening spaces. This aligns with previous research highlighting the relationship between community gardens and community connectedness through increased access to social opportunities as well as benefits to nutrition and sustainability [49, 50]. Local food systems can increase community member interactions through events and activities such as farmer’s markets and small local farms, which creates a stronger social network and resilience within the community [51]. Cardinia Shire, being a horticultural area, has the potential to foster these connections through farmers and local produce and build these farmer-to-community networks which have been shown to lead to stronger local food systems [51].

Participants in the current research highlighted their desire for more access to local produce to not only support community connection, but to support local retail owners and farmers, contribute to the local economy and to reduce food miles. Previous research has highlighted that small greengrocers are significantly cheaper than large supermarkets and have a large variety of produce [52]. However, current options for accessing this produce through small food retailers was seen as limited in Cardinia Shire, and therefore there was an overall reliance on large food retailers. The Australian food retail system is dominated by two large food retailers and has had a highly concentrated food retailer market compared to grocery markets in other developed countries [53]. Similar to the viewpoints in the present research, a study found Australian shoppers had intentions and desire to source and purchase Australian locallygrown food, but were restricted by time available as a commodity and further limited by oversaturation of large food retailers [54]. While some success of smaller independent food retailers has been seen within the context of the state of  TasmaniaAustralia, through a ‘shop local’ movement [55], the dominance of the large retailers continues to make it difficult for small independent food retailers within Australia.

The physical food environment of Cardinia Shire was amongst a range of triggers identified for experiencing food insecurity. Cost of living was discussed as a key driver for food budget amongst many participants, with mortgage and rent payments, petrol and other household bills being prioritised in budgeting. In Australia, food insecure households that receive government assistance payments have been found to be in significant financial stress, with energy and fuel being amongst the major budgetary stressors [56]. Additionally, the cost of both an ‘unhealthy’ or ‘healthy’ diet has been found to be unaffordable for Australians at the national poverty line [57]. Participants in the current study described the effects of the COVID-19 pandemic on their ability to access food from both a physical and financial perspective. Physically they did not have access to some of their usual affordable food procurement locations such as farmers and social enterprise markets. Additionally, the pandemic resulted in job loss or insecure employment affecting household income and therefore money available to purchase food. A sample of Australian women (*n* = 1005) showed 19.6% of households were experiencing food insecurity during the COVID-19 pandemic, indicative of an increase from pre-pandemic [14]. Additionally, a survey of people (*n* = 1170) from Tasmania, Australia showed 26% of the sample were experiencing food insecurity during the early stages of the COVID-19 pandemic [13]. Similar to the findings of the current study, these studies showed some of the predictors of this increase in food insecurity were job loss or job changes and poor mental health which may have been exacerbated by the pandemic [13, 14]. Post pandemic however, these determinates of food insecurity in particular cost of living pressures and mortgage stress are still continuing.

Regardless of the food security status of participants, coping strategies were used to ensure they had access to the food they needed for themselves and their household. At the core of many of these strategies were aspects of food literacy [45] such as planning, shopping for discounts and making use of limited ingredients. Not all participants discussed planning food shopping ahead of time but many mentioned budgeting in general to allow for adequate money for food. Shopping across multiple different food retailers and finding the cheapest options were discussed by participants across food security statuses. Previous research found, for recommended healthy diets to be affordable to those on minimum wage incomes or receiving welfare benefits, they would be required to only purchase the ‘cheapest option’ and even with this coping strategy, would struggle to afford the recommended diet [58]. Additionally, participants discussed their ability to cook multiple meal types to make use of these different and varying ingredients that were affordable at the time. In comparison, research by Begley et al. found food insecure individuals felt less confident in managing their money to buy healthy food, plan meals ahead of time and were less likely and less confident in cooking meals at home using healthy ingredients [59]. Proficient food literacy was described by participants in varying ways, however despite these apparent skills and knowledge healthy eating was not always able to be realised due to many environmental and financial barriers.

When the array of coping strategies to access food were no longer viable, participants experiencing food insecurity accessed food relief. While grateful for these services, participants highlighted a range of service structure and personal issues when accessing food relief services. Similar to previous findings, participants preferred not to access emergency food relief which was usually a last resort after trying other coping mechanisms however, were grateful for this ‘lifeline’ [60]. There was a lack of choice in where participants could access food relief services as well as the food that was available did not always align with their dietary needs, preferences, and expectations of quality. During the COVID-19 pandemic it was found that food relief providers did not have the ability to provide sufficient food to meet dietary recommendations for a balanced diet for those accessing their services [61]. The quality and quantity of food from food relief has been found to be inadequate, particularly to meet dietary and cultural needs [60, 62, 63]. Participants in the current study discussed the need for the services they are accessing to be grounded in dignity, fairness and respect, with issues such as having to prove their need before being allowed access also found in previous research [62]. Middleton et al. in their scoping review found that most participants felt the food relief services and volunteers they encountered to be supportive and the services were provided with dignity and respect [60]. However, there was still a sense of shame and stigma when accessing these services impacting dignity and feelings of inadequacy at being able to provide for themselves and their family [60, 63]. In Australia, approaches to address food insecurity have been focussed on emergency food relief and interventions that focus on food knowledge, skills and behaviour and are therefore, more short-term solutions [64]. This reliance on emergency food relief and charitable donations is not sustainable for these individuals and is not adequate to address the issue of ongoing food insecurity.

The findings of this study from those with lived experience highlights the potential for meaningful inclusion of community perspectives to be used to inform local government policy and program decisions [65]. Australian local governments are uniquely positioned and play a role across a wide range of activities in creating and supporting healthy, sustainable, and equitable local food systems. Recent calls in the literature by Carrad et al. [66] suggest “there is scope for more Australian local governments to adopt comprehensive, dedicated food system strategies that address health, sustainability, economic development and equity in an integrated way”. Informed by both international and national literature the 2022 Consensus Statement, ‘Towards a Healthy, Regenerative and Equitable Food System in Victoria’ [67] identifies potential leverage points that local governments and other important influencers of the food system can take collective action and collaborative policy making approaches to support equitable food systems. Drawing from their experience Cardinia Shire informed and contributed to the advocacy effort for this statement and is one local government who has signed a commitment to this consensus statement. Research reported in this study in conjunction with other local research undertaken in Cardinia Shire outlined in the introduction, fills a local knowledge gap in providing a deeper understanding of the true extent and experience of household food insecurity and factors that influence it at the municipal level. This collective research intelligence has contributed towards policy and practice change at the local (Cardinia Shire) and state-wide level, including advocacy for local services to support food security, local policy Cardinia Shire Council’s Liveability Plan and Community Food Strategy action particularly focused at local food growers and informing State Government food relief initiatives.

Using the novel method of photovoice this study explored the lived experiences of food access by people residing in Cardinia Shire. Additionally, the use of a mixed methods approach allowed for triangulation of findings and for the quantitative data to inform the qualitative interviews. This study used a convenience sampling strategy and therefore may not have reached those underrepresented groups such as culturally and linguistically diverse, low literacy and those with limited internet access. Due to COVID-19 pandemic lockdowns at the time of the photovoice interviews, all interviews were conducted via videoconferencing or telephone calls and therefore may have reduced comfort and rapport in discussion with the interviewer. However, attempts were made by interviewers prior and during the interview to build rapport with participants to aid with ease of conversation and use of participants' photographs allowed participants to discuss what they were comfortable with.

## Conclusions

While food choice is influenced by a range of determinants, the local food environment in which people live greatly impacts their food access and therefore food choices. A supportive local food system which promotes community connectedness and physical and economic access to local produce is crucial to support food security. The findings highlighted despite an array of participant skills to support food access for their households, the impact of external drivers that impact on money for food can challenge this realisation.

## Data Availability

The datasets generated and/or analysed during the current study are not publicly available due to the sensitive nature of some of the questioning and participants did not provide consent for information to be publicly available but are available from the corresponding author on reasonable request.

## Abbreviations

LGA: Local government area
USDA-HFSSM: United States Department of Agriculture Household Food Security Survey Module
